# Potential Role of *Lolium multiflorum* Lam. in the Management of Rice Weeds

**DOI:** 10.3390/plants9030324

**Published:** 2020-03-04

**Authors:** Sara Vitalini, Francesca Orlando, Valentina Vaglia, Stefano Bocchi, Marcello Iriti

**Affiliations:** 1 Department of Agricultural and Environmental Sciences, Università degli Studi di Milano, 20133 Milan, Italy; 2 Department of Molecular and Translational Medicine (DMMT), Università degli Studi di Brescia, 25123 Brescia, Italy; francesca.orlando@unibs.it; 3 Department of Environmental Science and Policy, Università degli Studi di Milano, 20133 Milan, Italy; valentina.vaglia@unimi.it (V.V.); stefano.bocchi@unimi.it (S.B.)

**Keywords:** Italian ryegrass, barnyard grass, rice, cover crops, organic farming, weed control, phytotoxic activity

## Abstract

The phytotoxic relationships between crops and weeds can cover a role in weed management, reducing the use of chemical herbicides. Starting from the organic farmers’ experience, the study aimed to define the inhibitory action of *Lolium multiflorum* Lam., used as a cover crop before rice sowing, against *Echinochloa oryzoides* (Ard.) Fritsch, one of the main rice weeds. In vitro 7-day assays were carried out in Petri dishes to compare the effect of different *L. multiflorum* Lam. parts, in the form of aqueous extract or powder, on the seed germination and seedling growth of *Oryza sativa* L. and *E. oryzoides* and to verify the hypothesis of a higher susceptibility of the weed. The total polyphenolic content, as the potential source of allelochemicals, in the *L. multiflorum* parts was measured. The results showed that both species suffer the phytotoxic action of *L. multiflorum*, but a more marked effect against *E*. *oryzoides* was recorded. In according with the polyphenol quantities, stem and inflorescence extracts showed the more significant species-specific inhibition. In all assays, the weed showed a stronger reduction in the root length and seedling vigor index, and, in some cases, also in the germination percentage and shoot length compared to rice.

## 1. Introduction

Weeds cause severe crop losses in rice production worldwide. The yield reductions in flooded paddy fields are due to the presence of invasive aquatic and semi-aquatic species. Among them, the species of the *Echinochloa* genus are the most common weeds in wetlands and water-saturated conditions and are included in the list of the ten worst weeds in the world [1]. In particular, *E. oryzoides* (Ard.) Fritsch, known as early watergrass, is a rice mimic with very similar emergence and flowering times that allow it to achieve greater competitiveness than other *Echinochloa* species by influencing rice in its early growth stages [2,3]. Therefore, any intervention able to control the incidence of this weed is useful to give rice a competitive advantage.

Rice growers in temperate regions (Europe, US, Australia) are particularly attentive to weed control. Within an established model of industrial agriculture, based on monoculture and high-input systems, they face no option other than the application of synthetic herbicides because of the low knowledge of alternative agronomic practices and plant-based solutions, as well as the unfeasibility of hand-weeding due to the high labor costs [4]. However, the herbicide-based weed management has proved to be unable to solve the issues, leading to well documented resistance phenomena as in the case of *E. oryzoides* [2,5,6].

In addition, the special attention of the European Union to the risks and hazards for humans, animals and the environment associated with the use of chemical substances has led to the banning in its member states of many herbicides, such as oxadiazon-based plant protection products, a compound largely used in rice fields [7,8,9].

Given the above, there is the need to move toward new and more sustainable weed management strategies [10,11].

In this context, the long-term experience of the organic farmers could be used to recognize and set innovative and good practices, transferable somehow also to the conventional or integrated systems [12,13]. They paid particular attention to solve the weed issue identified as the main cause of yield variability and loss in the Mediterranean regions, which is the main challenge for organic rice production [14,15].

Weed control in organic farming is carried out through crop rotation and the use of cover crops, smother crops and green mulching, which are important for regulating the weed seed population in the soil and the plant population in the field [16,17]. In this regard, several factors influence the weed growth such as competition for space, water and nutrients, changes in temperature and shade as well as toxic microbial products, soil pH and release of allelochemicals [18]. Especially allelopathy, defined as the release of compounds from the living or dead tissues of a plant species with strong phytotoxic effect towards another one, is a phenomenon which deserves attention in the study of alternative pest management options, thanks to its potential action in weed suppression [19,20]. It is known that some species or varieties, used as cover crop or crop in rotation, produce relevant amounts of allelochemicals significantly affecting the weed germination and growth [21].

Allelopathic compounds could be used to control weeds in both organic and conventional agriculture: in the first case, through the direct cultivation of allelopathic species, respecting the ban on the use of any products for herbicide purposes and with the principle of low-external-input farming [22]; while, in the second case, through the marketing and use of plant-based herbicides able to support and integrate weed management, reducing the need for synthetic herbicides.

Particularly for rice, a participatory research carried out by Orlando and co-workers [23] with a small group of organic farmers in North Italy identified a strategy based on the cultivation of *Lolium multiflorum* Lam. as the most promising practice for weed control. In the study area (Po Valley, between Piedmont and Lombardy regions), characterized by a typical Mediterranean climate, 94% of national rice production is concentrated. The farming systems are mainly based on continuous flooding and a wide use of pesticides and herbicides that caused the highest groundwater and surface water pollution in the country [24]. *L. multiflorum*, known as Italian ryegrass, is used by rice growers during the winter season as a cover crop. Then, in May, the rice is sown directly among the standing plants of *L. multiflorum* and, subsequently, its biomass is mowed, chopped or rolled, producing green mulch. The farmer’s empirical knowledge suggested to the researchers the existence of an allelopathic suppressive action of *L. multiflorum* versus *Echinochloa* spp., with a chemical inhibition of weed germination and growth, beyond the well-known competitive effect of green mulching. 

Accordingly, the present study was aimed to verify the inhibitory activity of different organs of *L. multiflorum* against *E. oryzoides*. Two in vitro bioassays were carried out in order to evaluate the possible release of phytotoxic chemicals from the cover crop separately from other factors occurring simultaneously in the field able to influence the weed growth and from the complex dynamics of the soil seed bank. The impact of the *L. multiflorum* biomass aqueous extracts and its powder was assessed versus the germination and seedling growth of both *E. oryzoides* and *O. sativa* to highlight a potential species-specific action of *L. multiflorum*.

## 2. Results

### 2.1. Stem Effects

The obtained data showed a significant impact of the *L. multiflorum* stem aqueous extract on all the considered indices, except for the mean germination time (MGT), in both target species, but with a more evident effect against *E. oryzoides* than *O. sativa*, (*p*-values ≤ 0.05 for the interaction “species *× L. multiflorum* stem extract treatment”) (Table 1).

In particular, the extract, from 20% to 100% concentration, significantly reduced the *E. oryzoides* germination percentage (by 50%–80%), while *O. sativa* germination was affected only by 100% extract concentration with a 36.4% decrease compared to the control. Moreover, stem extract was able to inhibit *E. oryzoides* root and shoot elongation (up to 93% and 57%, respectively) by significantly lowering the seedling vigour index (SVI) values for all used concentrations (*p*-value = 0.000). Otherwise, only the treatments with 50% and 100% extract concentrations were effective on *O. sativa* whose SVI, root and shoot length were reduced by 58%–83%, 53%–80% and 44%–57%, respectively. 

Bioassay carried out with stem powder provided less evident effects (Table 2). 

No significant results were detected for *O. sativa* in relation to the measured indices. Similarly, stems were not able to affect germination percentage and shoot growth of *E. oryzoides*. On the other hand, at 0.4 and 0.8 g/dm^2^, the treatment increased its MGT by 8% and decreased the root length up to 59% by significantly influencing SVI, reduced by 42% and 46%, respectively. In this case, the interaction “species × *L. multiflorum* stem powder treatment” was significant (*p*-values ≤ 0.05) only for root length and SVI. 

In general, the results obtained for the *L. mutiflorum* stems showed a higher susceptibility of the weed than the crop in their responses to the increasing concentrations (Table 1 and Table 2).

### 2.2. Inflorescence Effects

Similarly to the stems, *L. multiflorum* inflorescence extract affected the seed development of both studied species showing a greater inhibitory action on *E. oryzoides* compared to *O. sativa*, particularly on the three seedling growth parameters, namely SVI, root and shoot length (*p*-values ˂ 0.05 for the interaction “species × *L. multiflorum* inflorescence extract treatment”) (Table 3). Otherwise, there is no preferential effect by the extract in reducing the germination of the one of the two species.

Their germination percentage was remarkably lowered by 50% and 100% extract concentrations (*p*-values = 0.000). In the first case, the germinated seeds of *O. sativa* and *E. oryzoides* were 38% and 18%, respectively, while 100% extract concentration was able to completely inhibit them (0% germination). In addition, the 50% extract concentration decreased root length of both species by 83% and 92% than controls, as well as their shoot length (−33% and −70%) by significantly reducing the corresponding SVI values (−86% and −95%). Notably, the *E. oryzoides* shoot elongation was also affected by inflorescence 20% extract concentration (−26%). 

Like the extract, also *L. multiflorum* powdered inflorescences placed in direct contact with *O. sativa* and *E. oryzoides* seeds significantly affected all measured indices, except for MGT (Table 4). 

The germination percentage decreased by 21%–67% and 44%–78%, respectively; the root length by 80%–94% and 89%–97%, shoot length by 26%–56% and 21%–59%, SVI by 70%–94% and 79%–93%, due to both used quantities (0.4 and 0.8 g/dm^2^). The species showed a similar response to the treatments both as regards the germination percentage and the shoot length (*p*-values > 0.05). Accordingly, the interaction “species × *L. multiflorum* inflorescence powder treatment” was significant only for root length and SVI (*p*-values = 0.000) confirming the tendency towards greater susceptibility of *E. oryzoides* shown by the previous results.

### 2.3. Root Effects

Unlike stems and inflorescences, *L. multiflorum* root extract was not able to affect, at any used concentration, both MGT and germination percentage in the studied species. Furthermore, only 50% and 100% extract concentrations showed cases of significant impact on other considered indices (Table 5). 

At 100% extract concentration, a reduction by 27% was observed in the *O. sativa* and *E. oryzoides* shoot length (*p*-values ˂ 0.05) and by about 50% for their SVI values (*p*-values = 0.000). Roots decreased by 47% and 61% (*p*-values = 0.000), respectively.

Lastly, the significant interaction “species × *L. multiflorum* root extract treatment” with respect to root length and SVI (*p*-values ˂ 0.05), thanks also to the effect of the 50% extract concentration on the roots of *E. oryzoides* (−17%), showed a greater inhibition of the growth of weed seedlings compared to that of rice. 

The results of *L. multiflorum* root powder bioassay supported previous data on *E. oryzoides* showing that both used quantities (0.4 and 0.8 g/dm^2^) significantly influenced only its root elongation (decrease between 36% and 38% compared to the control) and SVI (decrease between 29% and 30%). Contrastingly, the powdered roots showed no effect against *O. sativa*, for which all the values of the measured indices were comparable to those of controls (Table 6). On the basis of these results, the interaction “species × *L. multiflorum* root powder treatment” was significant, showing a greater reduction in *E. oryzoides* root length and SVI. 

### 2.4. Seed Effects

The phytotoxic activity of *L. multiflorum* seeds was also assessed. Their aqueous extract impacted similarly on both target species that achieved growth values comparable to those of their controls for all considered indices (*p*-values > 0.05) (Table 7). The germination percentage of treated *E. oryzoides* and *O. sativa* was greater than 90%. MGT was the same as for untreated seeds while the root and shoot development showed insignificant differences as well as SVI values (*p*-values > 0.05). 

### 2.5. Polyphenol Content in L. multiflorum Extracts

**Figure 1:**
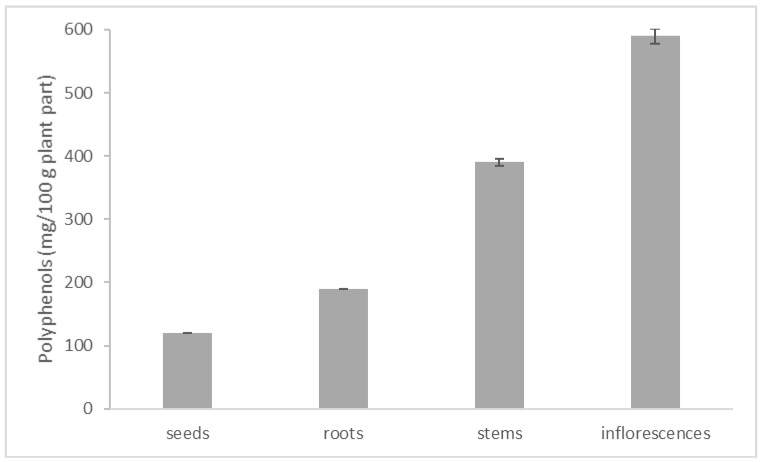
Total polyphenols detected in the aqueous extracts of the various *L. multiflorum* organs.

Figure 1 shows the polyphenol content in the aqueous extracts of the different investigated *L. multiflorum* parts measured using the Folin-Ciocalteau reagent. 

The highest content, equal to 590 mg GAE/100 g plant part, was identified in the inflorescences extract. Gradually lower quantities were found in stems (390 mg GAE/ 100 g), roots (190 mg GAE/ 100 g) and seeds (120 mg GAE/100 g), in accordance with the decreasing phytotoxic activity recorded for the various *L. multiflorum* parts against *E. oryzoides* and *O. sativa*.

## 3. Discussion 

Different studies reported the allelopathic activity of some *Lolium* species including *L. multiflorum* [25,26,27,28]. Usually, it is treated more as a weed capable of undermining the crop rather than as a crop cultivated with a function in the weed control and, therefore, in the crop protection. For example, Lehoczky and co-workers [26] described the inhibitory effects of aqueous extract obtained from *L. multiflorum* shoots on some of the main grown crops such as *Hordeum vulgare* L., *Triticum aestivum* L. and *Zea mays* L. However, other authors investigated the impact of decaying residues from *L. multiforum* used as a cover crop on *O. sativa* seedling development obtaining opposite results due to both inhibitory and stimulating effects [29,30,31]. To the best of our knowledge, very few data refer to the effectiveness of *L. multiflorum* against the weed growth [32]. Moreover, the relationship between *L. multiflorum* and *E. oryzoides* was never investigated. 

In this context, our results are particularly interesting and can be at the basis of the weed management strategies adopted by organic farmers who cultivated *L. multiflorum* before rice. 

*L. multiflorum* showed a preferential action with impacts significantly different and more severe on *E. oryzoides* rather than on *O. sativa*. Both *L. multiflorum* treatments, i.e., aqueous extracts and powder, obtained from all the investigated organs—inflorescences, stems and roots—showed a significant inhibitory effect on the weed. In particular, the stem and inflorescence aqueous extracts and the inflorescence powder significantly affected both seed germination and seedling growth, while the root aqueous extract and the stem and root powder reduced the root length. The root development of *E. oryzoides* showed always a greater reduction than those of *O. sativa*. In addition, species-specific phytotoxic effects were evident for the inflorescence and stem extracts, also regarding the shoot development and seed germination.

Additionally, *O. sativa* suffered the inhibitory effect of the aqueous extracts from *L. multiflorum*. In particular, inflorescence and stem extracts were able to reduce both the seed germination and the seedling growth (i.e., root and shoot elongation), while the root extracts affected only the seedling growth. On the other hand, the powder treatments showed minor activity and only those obtained from inflorescence had significant effect, inhibiting the seed germination and the seedling growth. 

Therefore, the data showed the existence of a phytotoxic activity by *L. multiflorum*, instead of its stimulating effects on rice, and, in general, a more marked action of the inflorescence, followed by stems and roots.

Finally, the seed aqueous extract was unable to affect *E. oryzoides* neither *O. sativa*. In both bioassays, all their growth parameters reached high values, similar to those of controls. The ineffectiveness of *L. multiflorum* seeds in influencing the development of other seeds could be attributed to the fact that the phytotoxic substances present in the cover crop are synthesized in a subsequent growth stage of the plant.

The preferential impact of *L. multiflorum* on the root development confirmed previous data documenting that the phytotoxic effect most observed in vegetative structures occurs on the root system [20]. Nevertheless, some studies on the relationships between species showed the different impact of the aqueous biomass extract on the measured variables. For example, Hoffman et al. [33] reported significant inhibition of root and shoot growth, without effect on germination, while Turk et al. [34] documented the decrease of germination and no reduction of the hypocotyl as well as Han et al. [35] recorded the inhibition of germination and root development but no effect on the shoots. On the other hand, the activity of phytotoxic compounds and their effects such as reduction in seed germination and seedling growth are caused by a variety of specific interactions and cannot been explained by just a single mode of action [28].

Lastly, polyphenols are a heterogeneous group of substances produced by the secondary metabolism of plants, where, in relation to chemical diversity, they play different roles. They can be simple low molecular weight compounds or complex structures conjugated with sugar moieties useful to plants for their structure, pigmentation, pollination, defense from predators and pathogens. Furthermore, their action as allelochemicals is known and investigated [36,37,38,39]. The different phytotoxicity of the investigated *L. multiflorum* organs could be partially related to the decreasing polyphenol values detected starting from the inflorescences.

Some allelopathic compounds were previously isolated from the aqueous leachates of decaying *L. multiflorum* residues. In particular, benzenepropanoic acid has proven to be effective in inhibiting the root and shoot growth of rice seedlings [29]. Other compounds such as caffeic acid, *p*-coumaric acid, ferulic acid and hydrocinamic acid were identified in the water fraction obtained from the fermentation of *L. multiflorum* shoots and roots. These phenolic acids seem to be responsible for the ability of the extract to reduce the shoot and root elongation in different rice cultivars [31]. Furthermore, the same type of extract was also able to affect the growth of two wheat cultivars [32].

In conclusion, the data obtained from our in vitro tests substantially confirmed the farmer empirical observations regarding the use of *L. multiflorum* as a cover crop, namely the corresponding reduction of *E. oryzoides* incidence, and explained the *O. sativa* poor density observed in certain fields under the same practice. In their opinion, *L. multiflorum* appears to negatively affect both the weed and rice development in the early growth stages, but *O. sativa* is less influenced than *E. oryzoides*, and on this thin difference it is possible to play for giving the crop a competitive advantage over the weed.

The farmers’ empirical knowledge comes from their direct long-time experiences in managing complex agro-ecosystems or are drawn from the rural tradition, thus including and safeguarding a stock of precious knowledge often fragmented or lost. It could be not a coincidence that in the past century, when the local farms combined the rice production with livestock, the cultivation of forage species such as *L. multiflorum* in rotation with rice was a common practice. Hence, the study results validate the usefulness of the farmers’ contribution in participatory research as a valuable guide for scientific inquiry and as a support for innovations in sustainable agriculture.

Moreover, *L. multiflorum* could be considered the starting point to formulate new plant-based and eco-friendly herbicides, functional to reduce the use of more dangerous synthetic compounds and the consequent environmental pressure due to agronomic practices in the rice area.

## 4. Materials and Methods 

### 4.1. Plant Material

Seeds of *O. sativa* (cv. Rosa Marchetti), *E. oryzoides* and *L. multiflorum* were obtained from ‘Terre di Lomellina’ organic farm located in the northern Italy (GPS coordinates: 45°10′28.329′′N 8°35′44.198′′E). They were stored at 4 °C until use after surface sterilization with 1% sodium hypochlorite by shaking for 7 min and repeatedly rinsing with distilled water. 

In the same farm, fresh plants of *L. multiflorum* were also collected. Their inflorescences, stems and roots were separately air-dried at room temperature (25 °C) in the shade and preserved in paper bags until extraction. 

### 4.2. Aqueous Extract Bioassay

The aqueous extract of each powdered part—inflorescences, stems and roots—of *L. multiflorum* was prepared mixing a suitable amount with distilled water (1:10, *w*/*v*) and shaking it at room temperature for 24 h. Afterwards, the mixture was filtered through gauzes to remove residues and centrifuged at 4500 rpm for 30 min. The obtained extracts were used as such (100%) and diluted with distilled water to give final concentrations of 1%, 10%, 20% and 50%.

Otherwise, in order to simulate the leaching from seeds, 60 unsterilized seeds were placed in 30 mL of distilled water on an orbital shaker, at room temperature, for 24 h. Subsequently, the obtained extract was filtered before use.

Ten sterilized seeds of *E. oryzoides* and *O. sativa* were sown into each Petri dish (90 mm diameter) containing 2 filter papers and 4 mL of each extract or its dilution were added. The same volume of distilled water was used as a control (0%). Petri dishes prepared in a vertical laminar flow hood and sealed with parafilm were kept in a growth chamber (25 °C/16 h light and 18 °C/8 h dark) for seven days. 

Concerning the inflorescence, stem and root extracts, five Petri dishes were realized for each combination of “species × *L. multiflorum* treatment”, according with the following randomized block design: two species (*E. oryzoides* and *O. sativa*) × six levels of concentration (100%, 50%, 20%, 10%, 1%, 0%) × three *L. multiflorum* organs (inflorescences, stems and roots) × five replicates. A similar experimental design with five repetitions was followed for the seed extract, considering only two levels of concentration (100% and 0%) and one *L. multiflorum* organ (seed). 

### 4.3. Plant Part Powder Bioassay

Different quantities (0.4 and 0.8 g/dm^2^) of each powdered part—inflorescences, stems and roots—of *L. multiflorum* were spread on two filter papers in Petri dishes (90 mm diameter). Afterwards, ten sterilized seeds of *E. oryzoides* and *O. sativa*, respectively, were placed and soaked with 5 mL of distilled water. The same volume of distilled water was used in the control samples (0 g/dm^2^ of powder). Petri dishes prepared in a vertical laminar flow hood and sealed with parafilm were kept in a growth chamber (25 °C/16 h light and 18 °C/8 h dark) for seven days. Five Petri dishes were realized for each combination of “species × *L. multiflorum* treatment” according with the following randomized block design: two species (*E. oryzoides* and *O. sativa*) × three levels of quantity (0.5 g, 0.25 g, 0 g) × three *L. multiflorum* organs (inflorescences, stems and roots) × five replicates.

### 4.4. Seedling Growth Parameter and Germination Indices

The number of germinated seeds in each Petri dish was recorded daily. At the seventh day, the length of their radicles and shoots was measured on graph paper under a stereomicroscope. The collected data were used to calculate the germination percentage, SVI [40] and MGT [41], respectively, by the following equations:

(1) $$\mathrm{Germination}\;\mathrm{Percentage}\;=\frac{Germinated\;Seed\;Number}{Seed\;Total\;Number}\times \;100$$

(2) SVI=(Mean Root Length+Mean Shoot Length)×Germination Percentage

(3) $$\mathrm{MGT}=\frac{\sum_{}^{}D_{}\times Germinated\;Seed\;Number}{\sum_{}^{}Germinated\;Seed\;Number_{}},$$

where *D* is the number of days from the beginning of germination.

### 4.5. Determination of Polyphenolic Content

The total polyphenolic content of the aqueous extracts was determined colorimetrically by the Folin-Ciocalteau method described by Scalbert et al. [42] with slight modifications. Briefly, 0.5 mL of each extract was added to 2.5 mL of 10% Folin-Ciocalteau reagent, previously diluted with distilled water. After 3 min, 2 mL of 7.5% sodium carbonate solution was added. The mixture was incubated in the dark for 1 h at room temperature and its absorbance was measured at 765 nm using a UV-vis spectrophotometer (Jenway 7205). A calibration curve was prepared with gallic acid standard solution at various concentrations (10 to 100 mg/L). The results were expressed as mg gallic acid equivalent (GAE)/100 g dry plant part. All the measurements were taken in triplicate and the mean values were calculated.

### 4.6. Statistical Analysis

The data were analyzed, with the support of IBM SPSS software, through the analysis of variance carried out separately for each bioassay (i.e., extract and powder bioassays) and *L. multiflorum* organs (i.e., inflorescences, stems, roots, seeds). The germination indices (i.e., germination percentage, SVI, MGT, root length, shoot length) measured for the two species (i.e., *E. oryzicola* and *O. sativa*) under different treatments were taken into account as dependent variables. 

The one-way ANOVA and the Turkey’s-b post hoc test were performed in order to establish the significant effect (at α ≤ 0.05) of the treatments with *L. multiflorum* (i.e., the different levels of concentration or quantity in extract and powder bioassay, respectively), on the species, and describe the homogenous subsets.

Moreover, the two-way ANOVA was performed, considering as factors the treatments with *L. multiflorum* and the species, in order to highlight the significant interaction (at α ≤ 0.05) between “species × *L. multiflorum* treatments”, and then highlighting the species-specific effects of the treatments and the different behaviors or susceptibility between the rice crop and the weed.

## Figures and Tables

**Table 1:** Germination indices measured for *E. oryzoides* and *O. sativa* under the effect of different concentrations of *L. multiflorum* stem extract.

Species	Stem Extract Concentration (%)	Germination (%)	MGT	Root Length (mm)	Shoot Length (mm)	SVI
*E. oryzoides*	0	100.0 ± 0.0 a	5.0 ± 0.0	56.2 ± 14.5 a	34.0 ± 3.9 a	9008 ± 1764 a
	1	83.2 ± 13.6 ab	5.8 ± 0.4	64.0 ± 4.0 a	28.0 ± 4.0 ab	6613 ± 657 b
	10	76.8 ± 17.4 ab	5.6 ± 0.5	62.0 ± 4.0 a	26.7 ± 0.6 b	5323 ± 227 b
	20	50.0 ± 35.6 bc	5.8 ± 0.5	58.0 ± 0.0 a	26.0 ± 0.0 b	2787 ± 0 c
	50	22.0 ± 22.8 c	5.0 ± 0.0	9.7 ± 6.7 b	22.0 ± 2.6 b	1198 ± 647 c
	100	20.0 ± 12.2 c	5.5 ± 0.6	4.0 ± 2.2 b	14.5 ± 1.0 c	482 ± 21 c
	F	13.756	2.892	36.382	22.701	43.232
	*p-*value	0.000 *	0.052	0.000 *	0.000 *	0.000 *
*O. sativa*	0	96.8 ± 4.6 a	5.0 ± 0.0	43.0± 13.5 a	19.8 ± 2.6 a	6072 ± 1525 a
	1	96.6 ± 3.5 a	5.0 ± 0.0	49.0 ± 1.0 a	19.0 ± 2.0 a	6364 ± 276 a
	10	96.6 ± 3.5 a	5.0 ± 0.0	52.0 ± 3.0 a	18.3 ± 5.5 a	6805 ± 1009 a
	20	96.6 ± 3.5 a	5.0 ± 0.7	47.0 ± 2.0 a	15.7 ± 1.5 ab	6042 ± 149 a
	50	79.0 ± 12.1 a	5.0 ± 0.0	20.2 ± 10.8 b	11.0 ± 1.6 bc	2564 ± 1235 b
	100	61.6 ± 21.1 b	5.2 ± 0.4	8.6 ± 2.1 b	8.6 ± 1.1 c	1003 ± 403 b
	F	9.922	0.286	19.413	15.045	24.718
	*p-*value	0.000 *	0.916	0.000 *	0.000 *	0.000 *
Interaction species × treatment						
	F	6.709	1.396	2.999	2.513	8.130
	*p-*value	0.000 *	0.252	0.025 *	0.05	0.000 *

Values are mean ± standard deviation, asterisk and different letters indicate statistically significant differences at *p*-value ≤ 0.05 among treatments in each species. F-value and *p*-value of the ANOVA test. MGT, mean germination time; SVI, seedling vigor index.

**Table 2:** Germination indices measured for *E. oryzoides* and *O. sativa* under the effect of different quantity of *L. multiflorum* powdered stems.

Species	Powdered Stem Guantity (g/dm^2^)	Germination (%)	MGT	Root Length (mm)	Shoot Length (mm)	SVI
*E. oryzoides*	0.00	100.0 ± 0.0	5.0 ± 0.0 a	92.7 ± 3.6 a	36.5 ± 1.5	12925 ± 371 a
	0.4	92.5 ± 9.6	5.3 ± 0.1 ab	43.1 ± 24.5 b	37.6 ± 3.7	7444 ± 2530 b
	0.8	95.0 ± 5.0	5.4± 0.3 b	38.4 ± 11.4 b	35.3 ± 2.9	6973 ± 1180 b
	F	1.964	7.226	20.427	0.780	23.126
	*p-*value	0.186	0.011 *	0.000 *	0.482	0.000 *
*O. sativa*	0.00	90.0 ± 10.0	5.0 ± 0.1	64.7 ± 3.9	27.6 ± 1.9	8297 ± 804
	0.4	90.0 ± 7.1	5.2 ± 0.1	55.8 ± 19.6	23.8 ± 4.1	7214 ± 2381
	0.8	88.8 ± 4.5	5.1 ± 0.1	54.3 ± 11.9	21.4 ± 5.4	6632 ± 1338
	F	0.118	2.000	0.878	2.923	1.321
	*p-*value	0.890	0.178	0.441	0.092	0.303
Interaction species × treatment						
	F	0.762	3.384	6.715	1.588	5.573
	*p-*value	0.478	0.052	0.005 *	0.227	0.011 *

Values are mean ± standard deviation, asterisk and different letters indicate statistically significant differences at *p*-value ≤ 0.05 among treatments in each species. F-value and *p*-value of the ANOVA test. MGT, mean germination time; SVI, seedling vigor index.

**Table 3:** Germination indices measured for *E. oryzoides* and *O. sativa* under the effect of different concentrations of *L. multiflorum* inflorescence extract.

Species	Inflorescence Extract Concentration (%)	Germination (%)	Mean Germination Time	Root Length (mm)	Shoot Length (mm)	Seedling Vigor Index
*E. oryzoides*	0	100.0 ± 0.0 a	5.0 ± 0.0	66.2 ± 14.5 a	34.0 ± 3.9 a	9008 ± 1764 a
	1	83.2 ± 7.5 a	5.6 ± 0.5	76.0 ± 1.0 a	33.3 ± 2.0 a	7733 ± 264 a
	10	90.2 ± 5.6 a	5.8 ± 0.4	68.0 ± 9.0 a	29.3 ± 2.5 ab	8405 ± 978 a
	20	94.8 ± 3.0 a	5.6 ± 0.5	62.0 ± 4.0 a	25.0 ± 0.6 b	9255 ± 772 a
	50	18.0 ± 21.7 b	5.3 ± 0.6	4.3 ± 0.6 b	10.3 ± 2.1 c	407 ± 276 b
	100	0.0 ± 0.0 c	n.d.	n.d.	n.d.	n.d.
	F	100.627	2.179	27.971	39.963	32.699
	*p-*value	0.000 *	0.113	0.000 *	0.000 *	0.000 *
*O. sativa*	0	96.8 ± 4.6 a	5.0 ± 0.0	43.0± 13.5 a	19.8 ± 2.6 a	6072 ± 1525 a
	1	96.8 ± 5.6 a	4.6 ± 0.5	44.3 ± 1.5 a	17.7 ± 0.6 a	5794 ± 508 a
	10	95.0 ± 5.4 a	4.6 ± 0.5	40.3 ± 3.5 a	18.3 ± 1.5 a	4825 ± 222 a
	20	88.4 ± 2.6 a	5.0 ± 0.7	48.7 ± 1.5 a	15.2 ± 4.1 ab	5806 ± 327 a
	50	37.0 ± 20.1 b	5.2 ± 0.4	7.2 ± 3.4 b	13.3 ± 2.5 b	825 ± 554 b
	100	0.0 ± 0.0 c	n.d.	n.d.	n.d.	n.d.
	F	101.674	1.385	21.825	3.155	27.790
	*p-*value	0.000 *	0.275	0.000 *	0.048*	0.000 *
Interaction species × treatment						
	F	1.39	2.764	4.45	17.641	4.656
	*p-*value	0.265	0.051	0.007 *	0.000 *	0.006 *

Values are mean ± standard deviation, asterisk and different letters indicate statistically significant differences at *p*-value ≤ 0.05 among treatments in each species. F-value and *p*-value of the ANOVA test. MGT, mean germination time; SVI, seedling vigor index.

**Table 4:** Germination indices measured for *E. oryzoides* and *O. sativa* under the effect of different quantity of *L. multiflorum* powdered inflorescences.

	Powdered Inflorescence Quantity (g/dm^2^)	Germination (%)	MGT	Root Length (mm)	Shoot Length (mm)	SVI
*E. oryzoides*	0.00	100.0 ± 0.0 a	5.0 ± 0.1	90.6 ± 5.9 a	36.2 ± 1.7 a	12688 ± 647 a
	0.4	56.4 ± 35.1 b	5.2 ± 0.2	9.8 ± 9.9 b	28.8 ± 3.7 b	2649 ± 627 b
	0.8	22.0 ± 31.9 b	5.6 ± 0.8	2.9 ± 0.8 b	14.9 ± 5.0 c	920 ± 57 c
	F	10.165	2.121	175.721	33.891	436.013
	*p-*value	0.003 *	0.182	0.000 *	0.000 *	0.000 *
*O. sativa*	0.00	92.0 ± 8.4 a	5.0 ± 0.0	63.2 ± 5.1 a	25.4 ± 1.3 a	8122 ± 355 a
	0.4	72.5 ± 10.9 b	5.1 ± 0.1	12.6 ± 8.3 b	18.8 ± 6.4 b	2401 ± 1489 b
	0.8	30.0 ± 12.2 c	5.0 ± 0.0	3.5 ± 1.1 c	11.3 ± 1.4 c	466 ± 223 c
	F	44.506	2.889	160.545	16.963	99.309
	*p-*value	0.000 *	0.095	0.000 *	0.000 *	0.000 *
Interaction species × treatment						
	F	2.292	3.357	15.724	2.014	21.465
	*p-*value	0.127	0.055	0.000 *	0.160	0.000 *

Values are mean ± standard deviation, asterisk and different letters indicate statistically significant differences at *p*-value ≤ 0.05 among treatments in each species. F-value and *p*-value of the ANOVA test. MGT, mean germination time; SVI, seedling vigor index.

**Table 5:** Germination indices measured for *E. oryzoides* and *O. sativa* under the effect of different concentrations of *L. multiflorum* root extract.

Species	Root Extract Concentration (%)	Germination (%)	MGT	Root Length (mm)	Shoot Length (mm)	SVI
*E. oryzoides*	0	100.0 ± 0.0	5.0 ± 0.0	66.2 ± 14.5 a	34.0 ± 3.9 a	9008 ± 1764 a
	1	93.2 ± 7.5	5.6 ± 0.5	68.3 ± 3.5 a	32.0 ± 1.0 a	8740 ± 120 a
	10	91.6 ± 2.6	5.6 ± 0.5	63.7 ± 1.5 a	32.3 ± 0.6 a	8787 ± 123 a
	20	93.2 ± 4.6	5.4 ± 0.5	67.0 ± 7.0 a	32.0 ± 2.0 a	9557 ± 537 a
	50	98.0 ± 4.5	5.2 ± 0.4	46.8 ± 4.0 b	32.2 ± 4.1 a	7734 ± 1040 a
	100	92.0 ± 8.4	5.4 ± 0.5	21.8 ± 2.9 c	24.8 ± 2.7 b	4321 ± 820 b
	F	1.870	1.171	22.461	5.315	14.805
	*p-*value	0.140	0.352	0.000 *	0.004 *	0.000 *
*O. sativa*	0	96.8 ± 4.6	5.0 ± 0.0	43.0± 13.5 a	19.8 ± 2.6 a	6072 ± 1525 a
	1	100.0 ± 0.0	5.0 ± 0.0	43.0 ± 4.0 a	20.7 ± 3.5 ab	6323 ± 752 a
	10	95.0 ± 5.4	4.6 ± 0.5	48.7 ± 1.5 a	25.3 ± 1.5 a	6920 ± 724 a
	20	94.8 ± 3.0	4.8 ± 0.4	47.6 ± 3.5 a	25.0 ± 2.0 a	6760 ± 579 a
	50	96.2 ± 3.3	5.0 ± 0.0	57.6 ± 6.0 a	26.4 ± 4.5 a	7784 ± 1184 a
	100	92.2 ± 6.1	5.0 ± 0.0	23.0 ± 6.5 b	14.4 ± 4.4 b	3304 ± 696 b
	F	1.867	1.680	10.479	7.595	10.370
	*p-*value	0.138	0.178	0.000 *	0.001 *	0.000 *
Interaction species × treatment						
	F	2.371	1.543	6.227	2.357	2.761
	*p-*value	0.59	0.201	0.000 *	0.06	0.033 *

Values are mean ± standard deviation, asterisk and different letters indicate statistically significant differences at *p*-value ≤ 0.05 among treatments in each species. F-value and *p*-value of the ANOVA test. MGT, mean germination time; SVI, seedling vigor index.

**Table 6:** Germination indices measured for *E. oryzoides* and *O. sativa* under the effect of different quantity of *L. multiflorum* powdered roots.

Species	Powdered Root Quantity (g/dm^2^)	Germination (%)	MGT	Root Length (mm)	Shoot Length (mm)	SVI
*E. oryzoides*	0.00	100.0 ± 0.0	5.0 ± 0.1	90.6 ± 5.9 a	36.2 ± 1.7	12688 ± 647 a
	0.4	94.0 ± 5.5	5.0 ± 0.1	57.9 ± 9.7 b	37.3 ± 1.4	8984 ± 1389 b
	0.8	94.0 ± 5.5	5.1 ± 0.2	56.2 ± 21.9 b	39.2 ± 3.9	8872 ± 1965 b
	F	3.000	1.471	9.275	1.704	11.394
	*p-*value	0.088	0.268	0.004 *	0.223	0.002 *
*O. sativa*	0.00	92.5 ± 8.3	5.0 ± 0.0	63.2 ± 5.1	25.4 ± 1.3	8166 ± 346
	0.4	92.0 ± 8.4	5.0 ± 0.1	62.7 ± 12.6	31.8 ± 4.5	8639 ± 1427
	0.8	90.0 ± 7.1	5.1 ± 0.1	52.7 ± 8.4	29.5 ± 5.3	7478 ± 1697
	F	0.139	1.600	2.063	3.205	1.015
	*p-*value	0.872	0.242	0.170	0.077	0.391
Interaction species × treatment						
	F	0.467	0.286	4.843	1.724	6300
	*p-*value	0.632	0.754	0.017 *	0.200	0.006 *

Values are mean ± standard deviation, asterisk and different letters indicate statistically significant differences at *p*-value ≤ 0.05 among treatments in each species. F-value and *p*-value of the ANOVA test. MGT, mean germination time; SVI, seedling vigor index.

**Table 7:** Germination indices measured for *E. oryzoides* and *O. sativa* under the effect of *L. multiflorum* seed extract.

Species	Seed Extract	Germination (%)	MGT	Root Length (mm)	Shoot Length (mm)	SVI
*E. oryzoides*	0%	96.7 ± 5.2	5.7 ± 0.4	42.0 ± 6.5	23.5 ± 3.0	6323 ± 785
	100%	91.7 ± 20.4	5.7 ± 0.1	50.8 ± 13.7	24.5 ± 7.2	6703 ± 1866
	F	0.338	0.037	2.034	0.086	0.212
	*p-*value	0.574	0.850	0.184	0.775	0.655
*O. sativa*	0%	90.0 ± 25.3	4.7 ± 0.1	51.4 ± 5.1	23.3 ± 5.1	5432 ± 2640
	100%	98.0 ± 12.1	4.7 ± 0.1	50.1 ± 3.6	21.8 ± 2.7	6040 ± 1308
	F	1.356	0.448	0.271	0.425	0.256
	*p-*value	0.271	0.519	0.614	0.529	0.624
Interaction species × treatment						
	F	1.640	0.146	2.301	0.387	0.024
	*p-*value	0.215	0.706	0.145	0.541	0.878

Values are mean ± standard deviation. F-value and *p*-value of the ANOVA test. MGT, mean germination time; SVI, seedling vigor index.
